# Cross-Sectoral Information Transfer in the Chinese Stock Market around Its Crash in 2015

**DOI:** 10.3390/e20090663

**Published:** 2018-09-03

**Authors:** Xudong Wang, Xiaofeng Hui

**Affiliations:** School of Management, Harbin Institute of Technology, Harbin 150001, China

**Keywords:** information transfer, Chinese stock sectors, effective transfer entropy, market crash

## Abstract

This paper applies effective transfer entropy to research the information transfer in the Chinese stock market around its crash in 2015. According to the market states, the entire period is divided into four sub-phases: the tranquil, bull, crash, and post-crash periods. Kernel density estimation is used to calculate the effective transfer entropy. Then, the information transfer network is constructed. Nodes’ centralities and the directed maximum spanning trees of the networks are analyzed. The results show that, in the tranquil period, the information transfer is weak in the market. In the bull period, the strength and scope of the information transfer increases. The utility sector outputs a great deal of information and is the hub node for the information flow. In the crash period, the information transfer grows further. The market efficiency in this period is worse than that in the other three sub-periods. The information technology sector is the biggest information source, while the consumer staples sector receives the most information. The interactions of the sectors become more direct. In the post-crash period, information transfer declines but is still stronger than the tranquil time. The financial sector receives the largest amount of information and is the pivot node.

## 1. Introduction

After decades of rapid growth, China has become the world’s second largest economy. It plays an important role in global trade. However, its stock market has displayed poor performance since the US subprime crisis. Under the background of deepening economic reform, the Chinese stock market began to boom around July 2014 [1]. Tens of millions of new investors entered the market. The great majority of them were retail investors, which tended to exhibit herd behavior. Moreover, many of these novice investors engaged in leveraged trading through various channels, for example margin financing of brokerages, shadow banking, or grey-market (over-the-counter, OTC) margin lenders [2,3]. Huge amounts of borrowed money flooded into the market [3]. The Shanghai stock exchange composite index (SSECI) soared from 2050.38 on 1 July 2014, to a peak of 5166.35 on 12 June 2015. It increased about 152% in just one year. However, after the peak, the market plunged drastically. From late June to late August of 2015, the SSECI declined about 40% [4]. It was one of the biggest falls in global stock market history [2]. In order to stabilize the market, the Chinese government took a series of actions, including organizing state-backed financial firms collectively called the “national team” to buy stocks directly, banning short sales, stopping new initial public offerings, etc. [3]. Through these efforts, the market turbulence ended in February 2016 [5]. This crash brought heavy losses to Chinese investors and the economy. Market capitalization up to trillions of US dollars evaporated [6]. It also impacted the world markets.

Analyzing information transfer is one of the fundamental subjects for complex system studies. It characterizes the interactions between components and provides important insights into the structure and dynamics of the system. This issue attracts many researchers from different fields, for instance neuroscience [7,8], physics [9,10], climatology [11,12], and zoology [13,14], etc. In econophysics, Kwon and Yang [15] analyzed the strength and direction of the information flow in 25 stock indices. They found that the US market was the biggest information source, while most information receivers are located in the Asian Pacific region. Yang et al. [16] used the annual gross domestic product (GDP) data of 27 Chinese provinces and autonomous regions to study the information transmission before and after the reform and opening up policy in 1978. The results showed that the policy promoted regional economy development and changed the influence of different areas. Dimpfl and Peter [17] analyzed the information transfer between US and European stock markets. They discovered that there existed bidirectional information flow. The US subprime crisis enhanced this information exchange. Sandoval [18] investigated the transfer of information in 197 of the largest financial companies in the world. The bank and insurance companies were found to play important roles in the transmission of information. Sensoy et al. [19] employed nine developing countries’ currency exchange rate and stock price data to research the exchange of information between them. The results suggested strong bidirectional information flow during the US subprime crisis. Bekios et al. [20] studied the information diffusion between commodity future and stock markets. The results indicated that finance, automobile, and energy stock sectors transmitted the most information to the commodity future market. Kim et al. [21] researched the information transfer in economy variables. It was discovered that Western countries had a strong influence in the world economic network. Japan’s influence decreased after the Asian currency crisis in 1997.

The aim of this paper is to apply effective transfer entropy (ETE) to research the information transfer in Chinese stock market around the crash of 2015, and to reveal the impacts of this crash on the interactions between sectors. To the best of our knowledge, this question has not been studied systematically in the existing literature. 

Transfer entropy (TE), introduced by Schreiber [22], is a very popular tool for measuring information transfer between time series. It has some remarkable properties. First, it is directional and can assess the direction of information. Second, it can be used in both linear and nonlinear environments. Third, it does not need specific model hypotheses. It is model-free and data-driven [23]. Because of these advantages, TE has been widely used in various domains [7,8,9,10,11,12,13,14,15,16]. In practice, the sample data is usually small and contains noise. To reduce these influences, Marschinski and Kantz combined a random shuffling procedure with TE and proposed ETE [24]. Lungarella et al. suggested that TE or ETE was the first choice when prior knowledge of the system is unknown [25]. In this paper, we consider the stock indices as continuous variables, avoiding information loss caused by data discretization. Since kernel density estimation (KDE) performs well in inferring probability density function [26,27], we adopt it to calculate ETE. The main contributions of this paper to the relevant literature are in three aspects: first, it analyzes the strength and scope of the information transfer in 10 Chinese stock sectors around the crash in 2015. Second, it applies node strength and betweenness centrality to assess sectors’ influences in different sub-periods. Third, it uses Chu-Liu-Edmond’s algorithm [28,29] to construct directed maximum spanning trees (MSTs) to research the backbones of the information transfer networks.

The rest of this paper is organized as follows. Section 2 introduces the methods. Section 3 describes the data and some preliminary analyses. Section 4 gives the results and some discussion. Section 5 concludes the paper.

## 2. Methodology

### 2.1. Transfer Entropy

Before introducing TE, we present the concept of Shannon entropy, which is fundamental for information theory. Let $R^{m}$ denote the *m*-dimensional real space and $A\in R^{m}$. Its Shannon entropy H(A) is defined as [30]:

(1) $$H(A)=-\int_{R^{m}}p(A)\log p(A)dA$$

where p(A) is the probability density function (PDF). Shannon entropy quantifies the amount of information that is needed to describe the variable, or the uncertainty of the variable. In this paper, the logarithm uses base 2; thus, the entropy is measured in bits.

Let another variable $B\in R^{n}$; the conditional entropy H(A|B) is [31]:

(2) $$H(A|B)=-\int_{R^{m+n}}p(A,B)\log p(A|B)dAdB$$

where p(A,B) and p(A|B) are the joint and conditional PDFs. They characterize the uncertainty of A given that B is known.

Given two stationary time series $X\in R^{1}$ and $Y\in R^{1}$, the TE from X to Y is defined as [32]:

(3) $$\begin{array}{lll} {TE}_{X\to Y} & = & H(y_{t+1}|y_{t}^{\left(k\right)})-H(y_{t+1}|y_{t}^{\left(k\right)},x_{t}^{\left(l\right)})\\ & = & \int_{R^{k+l+1}}p(y_{t+1},y_{t}^{\left(k\right)},x_{t}^{\left(l\right)})\log(\frac{p(y_{t+1}|y_{t}^{\left(k\right)},x_{t}^{\left(l\right)})}{p(y_{t+1}|y_{t}^{\left(k\right)})})dy_{t+1}dy_{t}^{\left(k\right)}dx_{t}^{\left(l\right)} \end{array}$$

where $y_{t}^{\left(k\right)}=(y_{t},y_{t-1},\ldots ,y_{t-k+1})$, $x_{t}^{\left(l\right)}=(x_{t},x_{t-1},\ldots ,x_{t-l+1})$ are the past states; $p(y_{t+1},y_{t}^{\left(k\right)},x_{t}^{\left(l\right)})$, $p(y_{t+1}|y_{t}^{\left(k\right)},x_{t}^{\left(l\right)})$, $p(y_{t+1}|y_{t}^{\left(k\right)})$ are the joint and conditional PDFs. ${TE}_{X\to Y}$ measures the uncertainty reduction or predictability improvement of $y_{t+1}$, which gains from $x_{t}^{\left(l\right)}$ that is not contained in $y_{t}^{\left(k\right)}$ itself [33]. In this way, it quantifies the predictive information transfer between variables [33,34]. By a simple transformation, Formula (3) can be rewritten as follows [31,35]:

(4) $${TE}_{X\to Y}=H(y_{t}^{\left(k\right)},x_{t}^{\left(l\right)})+H(y_{t+1},y_{t}^{\left(k\right)})-H(y_{t+1},y_{t}^{\left(k\right)},x_{t}^{\left(l\right)})-H(y_{t}^{\left(k\right)})$$

### 2.2. Effective Transfer Entropy

In practical applications, TE can produce spurious positive values for two independent time series because of limited data and random noise. ETE was proposed to reduce this bias. It is defined as [24,35]:

(5) $${ETE}_{X\to Y}={TE}_{X\to Y}-\frac{1}{M}\sum {TE}_{Xshuffed\to Y}$$

where $X_{shuffed}$ is the random shuffled series of X. M is the number of shuffles and it is set to 1000. In this paper, we first applied ${TE}_{Xshuffed\to Y}$ to test the significance of ${TE}_{X\to Y}$. Referring to References [27,33], if ${TE}_{X\to Y}$ is larger than the 95th percentile of ${TE}_{Xshuffed\to Y}$, ${TE}_{X\to Y}$ is considered significant nonzero, and the ETE is calculated according to Formula (5). Otherwise, it is considered that there is no transmission of information, and ETE is 0.

### 2.3. Kernel Density Estimation

According to Formulas (4) and (5), ETE can be calculated through Shannon entropy. Because the KDE-based method has good performance [26,27], we can apply it to compute ETE in this study.

Let $u_{1},u_{2},\ldots ,u_{N}$ be a sample of $U\in R^{d}$. Then, its PDF value $\hat{p}(u_{j})$ estimated by KDE with a kernel function K(⋅) is [26]:

(6) $$\hat{p}(u_{j})=\frac{1}{Nh^{d}}\sum_{i=1}^{n}K(\frac{u_{j}-u_{i}}{h})$$

where h is the bandwidth. In this paper we choose the Gaussian kernel, which is commonly used in practice. Therefore, Formula (6) can be written as [26,36]:

(7) $$\hat{p}(u_{j})=\frac{1}{Nh^{d}}\sum_{i=1}^{n}\frac{1}{\sqrt{{\left(2\pi \right)}^{d}\det(S)}}\exp(-\frac{{\left(u_{j}-u_{i}\right)}^{T}S^{-1}(u_{j}-u_{i})}{2h^{2}})$$

where S is the covariance matrix of the data; det(S) is the determinant of S.

The bandwidth is calculated by Formula (8), with reference to [26,36]:

(8) $$h{=(\frac{4}{d+2})}^{1/(d+4)}N^{-1/(d+4)}$$

After obtaining $\hat{p}(u_{i})$, the Shannon entropy can be computed by Formula (9) [37,38]:

(9) $$H(U)=-\frac{1}{N}\sum_{t=1}^{N}\log\hat{p}(u_{t})$$

where N is the length of the time series. 

Thus, the TE in Formula (4) can be estimated by Formula (10):

(10) $${TE}_{X\to Y}=-\frac{1}{N}(\sum_{t=1}^{N}\log\hat{p}(y_{t}^{\left(k\right)},x_{t}^{\left(l\right)})+\sum_{t=1}^{N}\log\hat{p}(y_{t+1},y_{t}^{\left(k\right)})-\sum_{t=1}^{N}\log\hat{p}(y_{t+1},y_{t}^{\left(k\right)},x_{t}^{\left(l\right)})-\sum_{t=1}^{N}\log\hat{p}(y_{t}^{\left(k\right)}))$$

Applying the above methodology, we calculate the ETE between two linear autoregressive processes [32]:

(11) $$X_{i+1}=\alpha X_{i+1}+\eta_{i}^{X};\;Y_{i+1}=\beta Y_{i+1}+\gamma X_{i}+\eta_{i}^{Y}$$

where $\eta^{X}$ and $\eta^{Y}$ are random numbers that obey standard normal distributions; α=0.5 and β=0.6. Let k=l=1. Then, the analytical value of ${TE}_{X\to Y}$ for the two processes is [32]:

(12) $${TE}_{X\to Y}=\frac{1}{2}\log\frac{\det C(Y_{i},X_{j})\det C(Y_{i+1},Y_{i})}{\det C(Y_{i+1},Y_{i},X_{i})\det C(Y_{i})}$$

where C(⋅) is the theoretical covariance matrix; det(⋅) denotes the determinant of a matrix. 

**Figure 1:**
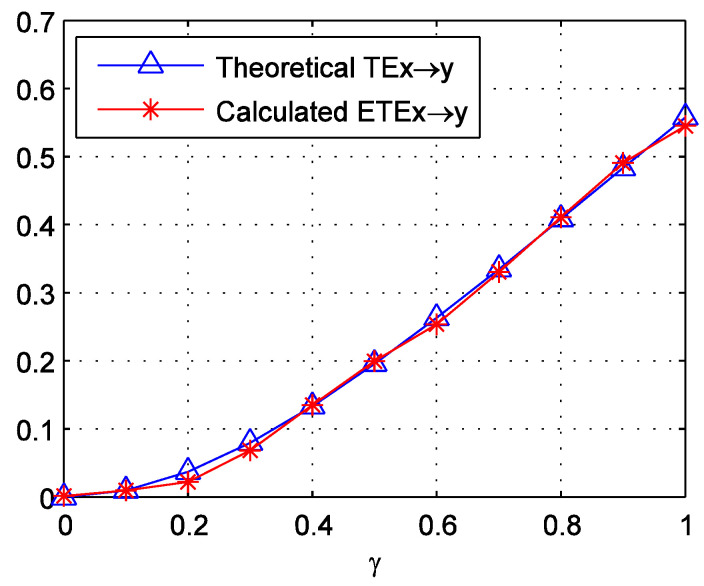
Theoretical ${TE}_{X\to Y}$ and calculated ${ETE}_{X\to Y}$ of the two linear autoregressive processes in Formula (11). γ increases from 0 to 1 with a step of 0.1.

For each γ, we generate 50 sample series of Formula (11) with a length of 200. This length is approximated to the sub-periods around the crash, which are divided in the later part of this paper. We then calculate the ETEs for these samples. The average values of these ETEs are displayed in Figure 1. It can be observed that the calculated ETEs match the theoretical TE well. The mean absolute error is just 0.0064. This supports the good performance of the methodology.

## 3. Data

According to the CICS Industry Classification issued by China Securities Index (CSI) Co., Ltd, all mainland China listed companies are divided into 10 first-level sectors. This paper therefore uses the daily closing price of the 10 CSI sector indices for the study. Their numbers and names are listed in Table 1. All data are downloaded from the WIND database, which is a leading Chinese financial information provider.

**Figure 2:**
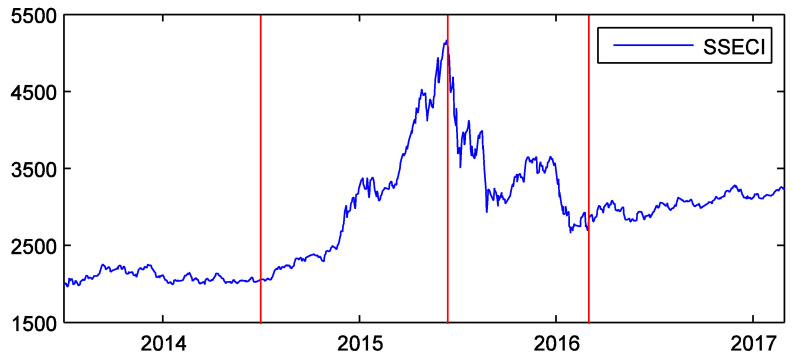
Shanghai stock exchange composite index (SSECI) and the four sub-periods around the time of the crash of 2015. The horizontal axis is the sample time ranging from 1 July 2013 to 28 February 2017. The red lines correspond to the dates of 1 July 2014, 12 June 2015, and 29 February 2016 from left to right.

The time range of the data is from 1 July 2013 to 28 February 2017. According to the market states, we divide the time into four sub-periods: the tranquil, bull, crash, and post-crash periods. This can help to analyze the influence of market states on the information transfer between sectors. The tranquil period extends from 1 July 2013 to 30 June 2014—approximately one year. During this period, the market was quite calm [1]. The bull period extends from 1 July 2014 to 12 June 2015. The market soared in this stage [3,39]. It reached the peak on 12 June 2015. After that day, it plunged drastically [3,39]. So, we take this day as the end of the bull period. The crash period starts on 15 June 2015 and ends on 29 February 2016. Because the dates of 13 June 2015 and 14 June 2015 fall on weekends, the start of the crash period is considered to be the latest trading day, 15 June 2015. Zhai analyzed the structural breaks of the Chinese stock market, and found that the crash of 2015 ended in February 2016 [40]. We therefore take 29 February 2016 as the end of the crash. This time is also in agreement with the literature [5,41]. The post-crisis period is from 1 March 2016 to 28 February 2017. It is also about one year. During this period, the market became stable again. Figure 2 shows the SSECI and the four sub-periods.

We apply Formula (13) to calculate the daily logarithmic returns of the 10 sector indices:

(13) $$R_{t}=\ln P_{t}-\ln P_{t-1}$$

where $R_{t}$ represents the logarithmic return; $P_{t}$ and $P_{t-1}$ denotes the price on day t and t−1, respectively.

Since TE needs the time series to be stationary, we apply the augmented Dickey-Fuller (ADF) test to examine the stationarity of the whole return series. The lag length is selected by Schwarz Information Criterion. The maximum lags are defined as 20. We also use the Jarque-Bera test to examine whether the whole return series obey Gaussian distribution. Table 2 shows the results.

From Table 2, it can be concluded that all 10 return series are stationary. However, they do not obey Gaussian distribution as a whole. In addition, we conducted the ADF and Jarque-Bera tests on the four sub-periods of the return series. The results of the ADF test are all significant at the 1% level. This indicates that the series are also stationary in the four sub-periods. However, the results of Jarque-Bera test are mixed at the 1% significance level. Some are significant and some are not.

We use the autocorrelation function to determine the delay time k and l. The lag of its first zero-crossing, or the lag required for the function to decrease to 1/e, can be selected as the delay time [42,43]. The results show that the first zero-crossings of all sectors’ autocorrelation functions are between 1 and 2, and the functions’ values of lag 1 are already below 1/e. We therefore set k=l=1 in this paper. This indicates the weak memory of the daily stock returns. This configuration is in accordance with the literature [15,18,20,44,45].

## 4. Results and Discussion

### 4.1. ETE between Sectors

**Figure 3:**
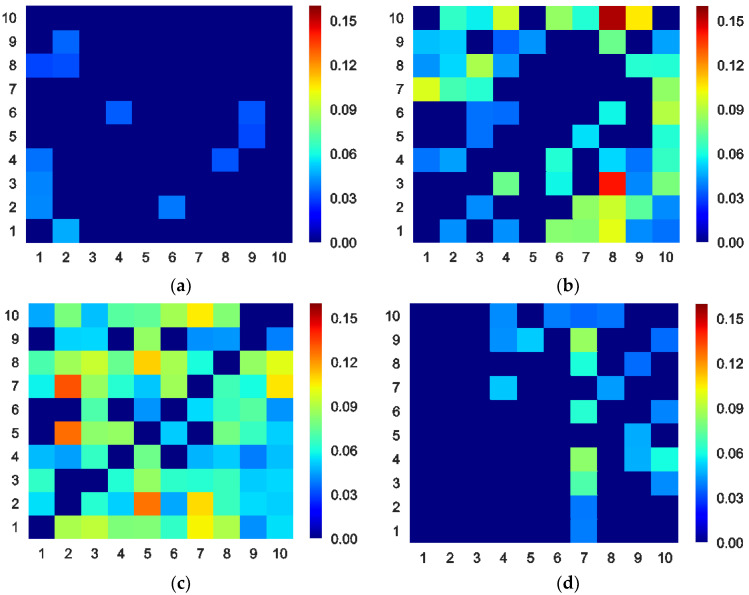
Colormaps of the effective transfer entropy (ETE) between the 10 sectors during the (**a**) tranquil, (**b**) bull, (**c**) crash, and (**d**) post-crash periods. The range of the colorbars is from 0 to 0.1533.

We calculate the ETEs between 10 Chinese stock sectors during the four sub-periods. In order to visualize and compare them conveniently, we use colormaps to display them. All colorbars are adjusted to the same range. Figure 3 and Table 3 shows these colormaps and the mean ETE of each sub-period. The direction of the ETE is from the vertical axis to the horizontal axis. The numbers on the axes are the serial number of the sectors in Table 1. From Figure 3 and Table 3, it can be observed that the information transfer exists in only a few sectors in the tranquil period, and that its strength is weak. This indicates feeble interactions between sectors in this stage. In the bull period, the strength and scope of the information transfer increases, suggesting stronger interactions. In Figure 3b, there are two high ETE blocks with the coordinates (8, 3) and (8, 10). This means that the industry (No. 3) and the utility (No. 10) sector indices transmit much information to the information technology sector index (No. 8). One of the possible reasons for this is that during the booming market phase, informatization construction in the industry and the utility fields (for example, the state-prompted smart city, intelligent grid, “Made-in-China 2025”, and “Internet-plus” plans [46,47,48,49]) provides huge demands and opportunities for information technology companies. This potential economical link may enhance the information transfer [50]. In the crash period, the strength and scope of the information flow increases further, and it reaches the maximum of the four sub-periods. This implies the strongest interactions between sectors. In the post-crash period, the market becomes stable again. The information transfer weakens, but it is still stronger than the tranquil period. The scope of the information transfer is also different from that in the tranquil stage.

From the perspective of market microstructure, the movement of the stock price is determined by the arrival of new information, and by the process that absorbs the information into the price [51]. According to the Efficient Market Hypothesis, if the market is perfectly efficient, the price reflects all current information. Newly arrived information is incorporated instantaneously into the price. In this ideal condition, there is no predictability and information transfer between the stocks [52,53]. However, researchers have discovered that market frictions widely exist in capital markets; for instance, the limited attention of investors, asymmetric information, and noise traders, etc. [54]. They cause a difference in the speeds of the information absorption of prices, and result in the predictability and information transfer from the faster one to the slower one [50,54,55]. In practice, bidirectional information transfer can be seen. This is because different information may coexist in the market. Different stock may react to different information at different speeds. On the other hand, predictability can be an important indicator of market efficiency [56,57,58]. From this aspect, it can be inferred that the market efficiency in the tranquil period is relatively high. It deteriorates in the bull time, and it is the worst in the crash period. In the post-crash stage, it obtains some recovery. Contrasting with the drastic boom and crash of the stock market, the macroeconomic variables of China are steady. We conclude that their effect on the change of market statuses and ETE is weak in these periods. This conclusion is line with Song [2]. He applied multifactor models to examine the effect of macroeconomic variables and found that the bull market was not sensitive to the macroeconomic variables.

### 4.2. Centrality of Sectors

For the further study of the sectors’ interactions, we construct the information transfer network. The sectors are considered as the nodes in the network. If there is a nonzero ${ETE}_{i\to j}$, a directed edge is added from sector i to sector j, with the weight of ${ETE}_{i\to j}$. Then we obtain the network. Node strength is a common centrality measure. For a weighted directed network, it can be divided into Out Node Strength ${NS}_{out}$ and In Node Strength ${NS}_{in}$:

(14) $${NS}_{out}^{i}=\sum_{j}{ETE}_{ij};\;{NS}_{in}^{i}=\sum_{k}{ETE}_{ki}$$

Out Node Strength reflects the influence of a node on others. In Node Strength measures the influence of a node receiving from others [59]. Table 4 and Table 5 display the Out and In Node Strengths of the sectors. The largest values are in bold. In the tranquil period, we can observe that the Out and In Node Strengths of the sectors are small. The energy (No. 1) and material (No. 2) sectors have relatively larger In Node Strengths than others. According to Cohen and Shahrur [50,60], the stock prices of downstream companies usually lead the upstream companies’ stock prices. In supply chains, energy and material companies are usually upstream. It may therefore cause the two sectors to receive relatively more information. In the bull period, the Out and In Node Strengths all increase. The utility sector (No. 10) has the largest Out Node Strength. China has invested heavily in the infrastructure construction for years. There are great opportunities in this field. A booming market enhances investors’ confidence. The utility sector, which is an important domain of infrastructures, may attract significant attention from investors. This could cause the utility sector to react fast to information and output more information than others in this period. The information technology (No. 8) sector receives the most information, indicating that it is affected greatly by other sectors. In the crash period, apart from the Out Node Strength of the utility sector, which exhibited a slight decrease, other sectors’ values continue to increase. The information technology sector outputs the largest amount of information in this time. Since the herding activities in this sector are found to be stronger than others [61] and information technology companies usually have a high price-earnings (P/E) ratio, it may make this sector more sensitive to market turmoil. Meanwhile, the financial (No. 7) and energy sectors also have relatively strong Out Node Strengths. In this period, except for the utility sector, others’ In Node Strengths all grow. The consumer staples (No. 5) sector receives the most information. Companies in this sector usually produce essential commodities in people’s daily lives. This may make it less sensitive to market turbulence, and it results in receiving relatively more information from other sectors. In the post-crash period, the Out and In Node Strengths of the sectors all decline. The telecommunication service (No. 9) sector outputs the most information. The In Node Strength of the financial sector is much larger than others, indicating that it is heavily impacted by other sectors. This is because many large financial firms are part of the “national team”, which aims to buy huge amount of stocks during the crash time in order to stabilize the market [3]. These financial firms therefore become the stakeholders and important money providers for many companies in different sectors. Other sectors’ statuses therefore may influence the financial condition of these financial firms, as well as the investors’ moods and strategies. This further impacts the price of these financial companies.

Betweenness is another centrality measure. It quantifies a node’s underlying ability to control the information flow in the network [62]. Its definition is based on the number of shortest paths between nodes. For a weighted directed network, the shortest path $d_{ij}^{w}$ between node i and j is [62]:

(15) $$d_{ij}^{w}=\min(\frac{1}{w_{ih_{0}}}+\frac{1}{w_{h_{0}h_{1}}}+\cdots +\frac{1}{w_{h_{k}j}})$$

where $v_{h_{0}},v_{h_{1}},\cdots ,v_{h_{k}}$ are the intermediary nodes on the path from node $v_{i}$ to $v_{j}$. The shortest path can be derived using Dijkstra’s algorithm and the weighted betweenness centrality (WBC) is defined as [62]:

(16) $$WBC(i)=\sum_{i\neq s,i\neq t,s\neq t}\frac{g_{st}^{w}(i)}{g_{st}^{w}}$$

where $g_{st}^{w}$ is the number of the shortest paths from node $v_{s}$ to $v_{t}$. $g_{st}^{w}(i)$ is the number of the shortest paths from $v_{s}$ to $v_{t}$ that also pass through $v_{i}$.

**Figure 4:**
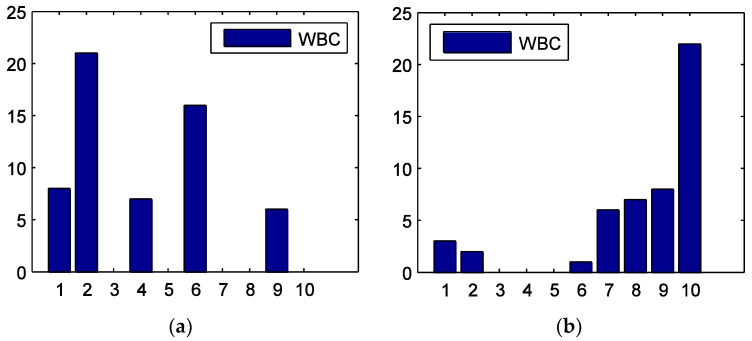

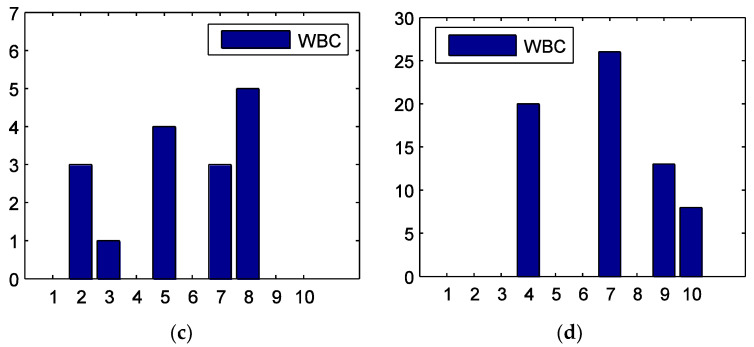
Weighted betweenness centrality (WBC) for the 10 stock sectors during the (**a**) tranquil, (**b**) bull, (**c**) crash, and (**d**) post-crash periods. The horizontal axis is the number of the sectors.

Figure 4 shows the WBCs of the 10 sectors in the four sub-periods. In the tranquil period, the material (No. 2) sector has the largest WBC. In the bull period, the utility (No. 10) sector has the largest WBC, while other sectors have much smaller values. This implies that the utility sector is the hub node, and it is very important for the information transmission in this stage. In the crash period, the WBCs are generally smaller. This is because in this phase, the information flow grows and sectors have more routes to connect with each other. The information technology (No. 8) sector has a relatively larger value. In the post-crash period, the financial sector (No. 7) has the largest WBC and is the pivot node of the information flow.

### 4.3. Directed Maximum Spanning Tree

The MST is one of the spanning trees of a network with the maximum total edge weights. It can help to disentangle the network and visualize the key structures [63]. For the undirected network, the MST can be built using the algorithms of Kruskal or Prim. However, in this paper, the information transfer network is a directed one. Thus, we adopt Chu-Liu-Edmond’s algorithm [28,29] to build the directed MST. It is also called the maximum arborescence, and is the backbone of a network [29]. 

**Figure 5:**
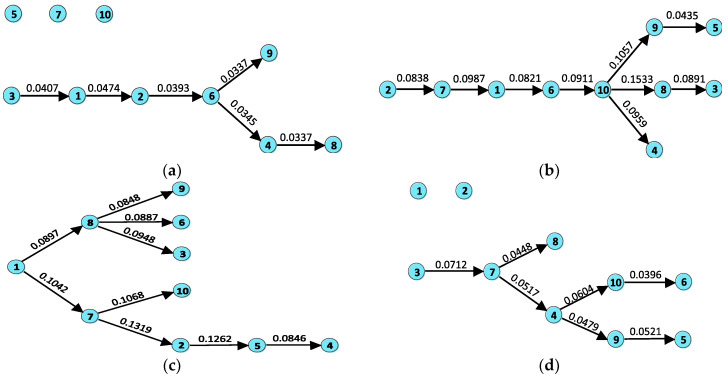
Directed maximum spanning trees (MSTs) of the 10 stock sectors during the (**a**) tranquil, (**b**) bull, (**c**) crash, and (**d**) post-crash periods. The values on the edges are their weights.

Figure 5 shows the directed MSTs in the four sub-phases. It can be seen that the structures of the trees are very different. This indicates that the market state has heavy impacts on the structure of the information transfer network. It also implies that information transfer can be a potential indicator for the market status. From Figure 5, we can observe that there are isolated sectors in both tranquil and post-crash periods. These isolated sectors have weak interactions with others, indicating that they could be references for portfolio diversification. In the bull and crash periods, the stronger information flow leads to the improvement of connectivity of the network. All sectors are contained in the trees. This means that the opportunity for asset diversification declines. Let the number of hops from the root node to itself be 0, so that the average hops of nodes to the root is 3.7 in the bull period. However, the value decreases to 1.9 in the crash time, suggesting that the tree has a more compact structure and the interactions between sectors are more direct in the crash stage.

## 5. Conclusions

Using ETE and the 10 sectors’ data from July 2013 to February 2017, this paper studies the information transfer in the Chinese stock market around its crash in 2015. According to the market states, the time range is divided into four sub-periods: the tranquil, bull, crash and post-crash periods. The stock data is considered as a continuous variable to avoid the subjectivity and information loss induced by data discretization. The KDE method is applied to compute the ETEs between sectors. Then, the information transfer network is constructed based on the ETEs. The influences of the sectors are analyzed by centrality measures. Lastly, a directed MST is used to disentangle the network.

The results display that the information transfer between sectors is weak in the tranquil period. The energy and material sectors are the main information receivers. In the bull period, the strength and scope of the information transmission both increase. The utility sector outputs much information, and it is the hub node. The information technology sector receives the most information in this stage. In the crash period, the information flow continues to grow, and it reaches the maximum of the four sub-periods, indicating the worst market efficiency. The information technology sector outputs the most information in this phase. The consumer staples sector is the biggest information receiver. The directed MST has a compact structure, suggesting more direct interactions between sectors. In the post-crash period, the strength and area of the information flow decreases, but it is still larger than that in the tranquil period. The telecommunication service sector emits the most information in this stage. The financial sector receives the largest amount of information, and it is the pivot node for the information transmission. 

These findings can help us to understand the structure of the Chinese stock market. As information transfer reveals the predictability of the market, this study can also provide references to investors for the selection of investment strategies.

## Figures and Tables

**Table 1:** The numbers and names of the 10 China Securities Index (CSI) sector indices.

No.	Index Name	No.	Index Name
1	CSI Energy	6	CSI Health Care
2	CSI Materials	7	CSI Financials
3	CSI Industrials	8	CSI Information Technology
4	CSI Consumer Discretionary	9	CSI Telecommunication Services
5	CSI Consumer Staples	10	CSI Utilities

**Table 2:** Results of the augmented Dickey-Fuller (ADF) and Jarque-Bera tests for the whole return series of the 10 sectors.

No.	ADF Statistic	Jarque-Bera Statistic	No.	ADF Statistic	Jarque-Bera Statistic
1	−28.6154 ***	765.3783 ***	6	−28.4199 ***	902.8109 ***
2	−28.2453 ***	823.1612 ***	7	−28.7902 ***	953.3962 ***
3	−26.7147 ***	783.1250 ***	8	−27.1013 ***	351.2834 ***
4	−27.7279 ***	753.9022 ***	9	−27.6364 ***	548.0041 ***
5	−23.0576 ***	867.0495 ***	10	−27.9620 ***	970.4217 ***

Note: *** denotes statistical significance at the 1% level.

**Table 3:** Average ETE between sectors in the four sub-periods

	Tranquil	Bull	Crash	Post-crash
Average ETE	0.0049	0.0384	0.0619	0.0123

**Table 4:** Out Node Strengths of the 10 sectors in the four sub-periods.

No.	Sector Name	Pre-Bull	Bull	Crash	Post-Crash
1	Energy	0.0474	0.4301	0.7006	0.0396
2	Materials	**0.0811**	0.3366	0.6279	0.0392
3	Industrials	0.0407	0.3985	0.5149	0.1133
4	Consumer Discretionary	0.0714	0.3055	0.4283	0.1918
5	Consumer Staples	0.0316	0.1557	0.5451	0.0471
6	Health Care	0.0682	0.2251	0.3525	0.1048
7	Financials	0	0.3147	0.7130	0.0965
8	Information Technology	0.0628	0.3573	**0.7729**	0.0989
9	Telecommunication Services	0.0361	0.3045	0.3194	**0.2176**
10	Utilities	0	**0.6259**	0.5983	0.1565

**Table 5:** In Node Strengths of the 10 sectors in the four sub-periods.

No.	Sector Name	Pre-Bull	Bull	Crash	Post-Crash
1	Energy	**0.1509**	0.2314	0.3450	0
2	Materials	0.1156	0.3289	0.6136	0
3	Industrials	0	0.3278	0.6609	0
4	Consumer Discretionary	0.0345	0.3319	0.4932	0.1367
5	Consumer Staples	0	0.0435	**0.7335**	0.0521
6	Health Care	0.0393	0.2888	0.4924	0.0396
7	Financials	0	0.2835	0.5885	**0.4808**
8	Information Technology	0.0337	**0.6807**	0.6157	0.0832
9	Telecommunication Services	0.0653	0.3646	0.4743	0.1325
10	Utilities	0	0.5725	0.5558	0.1806
